# The relationship between background parenchymal enhancement, amount of fibroglandular tissue and synchronous contralateral breast cancer in preoperative magnetic resonance imaging of newly diagnosed breast cancer patients

**DOI:** 10.1186/s12880-026-02402-3

**Published:** 2026-05-20

**Authors:** Yue Yang, Chungen Wu, Dan Wang, Mingliang Wang, Zhuhua Zhu, Xiaoer Wei, Wenbin Li

**Affiliations:** Institute of Diagnostic and Interventional Radiology, Shanghai Sixth People’s Hospital Affiliated to Shanghai Jiao Tong University School of Medicine, No. 600, Yi Shan Road, Shanghai, 200233 China

**Keywords:** Background parenchymal enhancement, Fibroglandular tissue, Synchronous contralateral breast cancer, Breast cancer, Magnetic resonance imaging

## Abstract

**Objective:**

This study aims to evaluate whether the amount of fibroglandular tissue (FGT) and the level of background parenchymal enhancement (BPE) on magnetic resonance imaging (MRI) are associated with synchronous contralateral breast cancer (CBC) during preoperative MRI staging of newly diagnosed breast cancer patients.

**Materials and methods:**

From January 2010 to December 2019, core needle biopsy-confirmed newly diagnosed breast cancer patients who underwent preoperative bilateral breast MRI were screened. 61 eligible patients with pathologically proven synchronous CBC were enrolled as the study group, while 122 matched primary breast cancer patients without synchronous CBC were selected as the control group.Odds ratios (ORs) and corresponding 95% confidence intervals(CIs) for BPE and FGT as predictors of synchronous CBC were analyzed by conditional logistic regression. The factors with significant differences between the study group and the control group were further analyzed by performing receiver operating characteristic (ROC) curve analysis to determine the area under the curve (AUC), sensitivity, and specificity. BPE and FGT were independently evaluated from MRI scans by Reader 1 and Reader 2, and their findings were statistically analyzed independently. The DeLong test was used to compare the statistical significance of AUC differences between Reader 1 and Reader 2.Kappa analysis was used to calculate the level of agreement between their findings.

**Results:**

BPE level was associated with synchronous CBC. Compared to women with minimal or mild BPE, those with moderate or marked BPE were more likely to have synchronous CBC (Reader 1: OR = 2.90, *P* < 0.01; Reader 2: OR = 3.36, *P* = 0.02). ROC showed the effectiveness of BPE level in differentiating contralateral CBC patients from control subjects with reader 1 (AUC 0.66, sensitivity 67.2%, specificity 63.1%) and reader 2 (AUC 0.67, sensitivity 80.3%, specificity 53.3%). No statistically significant difference in AUC was detected between Reader 1 and Reader 2 (DeLong test: 95% CI: -0.03–0.06; Z = 0.823, *P* = 0.410).No correlation was found between the amount of FGT and synchronous CBC. Further, the kappa values indicated substantial agreement between Reader 1 and Reader 2 with regard to the BPE and FGT values.

**Conclusion:**

High BPE level shows a correlation with an increased risk of synchronous CBC in newly diagnosed breast cancer patients; however, this association does not establish independence and could be influenced by unmeasured confounders. Thus, BPE alone cannot guide clinical decisions.

**Supplementary Information:**

The online version contains supplementary material available at 10.1186/s12880-026-02402-3.

## Introduction

There is a growing interest in biological factors that induce malignant transformation of breast tissue in women. While some clinical and genetic risk markers have been identified, imaging-based biomarkers have been receiving more attention in recent years [1]. Mammographic density has been shown to be a biomarker for predicting breast cancer risk: Women with mammographically dense breasts have a higher risk of breast cancer [2–5]. The amount of FGT derived from volumetric MRI images could be used to indicate the breast density [6]. Background parenchymal enhancement (BPE) refers to the enhancement of normal breast tissue on contrast-enhanced MRI, and it is usually symmetric and bilateral [6, 7]. Many studies have shown that the BPE and FGT values based on MRI are strongly associated with the risk of breast cancer, and they are considered as independent biomarkers of breast cancer risk [6, 8–10]. Some studies have put forward different views. Some argue that a high level of BPE is not necessarily a smoking gun for an increased risk of breast cancer [11].Until recently, a meta-analysis showed that BPE can predict breast cancer in high-risk populations, but in average-risk populations, BPE only represents the biological behavior of normal breast tissue and may not be associated with the development of breast cancer [12].

As a high-risk group of breast cancer, the risk of contralateral primary breast cancer in patients with unilateral breast cancer (UBC) is 4–6 times higher than that in the general population [13]. Many recent studies have begun to explore the relationship between imaging biomarkers and contralateral breast cancer (CBC) risk [14–16]. Emerging data show that mammographic density is a risk indicator for CBC in women with primary breast cancer. Patients with dense breasts had a significantly higher risk of CBC development than those with non-dense breasts (odds ratio [OR] = 1.80) [15, 16].

However, there is no study on risk prediction of CBC based on BPE and FGT measured on MRI. Furthermore, recent studies suggest that FGT, as a biomarker of breast cancer risk prediction, is only effective in short-term prediction in high-risk population [17]. Therefore, based on these previous findings, the purpose of this study is to retrospectively analyze whether FGT and BPE determined from MRI scans are associated with synchronous CBC during the preoperative MRI for UBC.

## Materials and methods

### Ethical approval

This retrospective study was approved by the Ethics Committee of Shanghai Sixth People’s Hospital and obtained informed consent from all participants. This study was conducted in accordance with the Declaration of Helsinki.

Study design and patient selection.

Between January 1, 2010, and December 30, 2019, 2896 consecutive patients newly diagnosed with breast cancer via core needle biopsy pathology at our hospital underwent preoperative bilateral breast MRI.The level of suspicion of malignancy of the contralateral lesion on MRI was determined according to the ACR BI-RADS^®^ Atlas Breast Imaging Reporting and Data System [18, 19]. According to the guidelines, core needle biopsy or surgical excision biopsy was performed in patients with contralateral BI-RADS 4 or 5 lesions; follow-up was recommended for those with contralateral BI-RADS 3 lesions [18, 19]. The pathological findings for 78 of these patients were primary breast cancer in the contralateral breast. These patients were diagnosed as synchronous CBC. This study included these patients with synchronous CBC, and excluded patients who had a history of radiotherapy, chemotherapy or hormone replacement therapy, bilateral oviduct oophorectomy, or who had undergone contralateral breast core needle biopsy within 2 weeks before the MRI examination due to post-biopsy breast edema [7, 20]. Therefore, out of the 78 women with synchronous CBC, 61 patients were enrolled in the study group(synchronous CBC cases).

The control group was selected from patients with primary breast cancer without contralateral cancer, matched to the study group at a 1:2 ratio based on age (± 1 years), histological subtypes(lobular carcinoma, ductal carcinoma, or other subtype such as tubular carcinoma), and the depth of invasion(carcinoma in situ or invasive carcinoma) [21, 22]. Randomized screening was performed using an electronic medical record system (Tech-winning, Shanghai, China) for case storage, extraction and screening. Patients with a history of radiotherapy, chemotherapy or hormone replacement therapy, bilateral oviduct oophorectomy, or who had undergone contralateral breast core needle biopsy within 2 weeks before the MRI examination were excluded [7, 20]. The final control group included 122 (UBC cases) female patients.

In cases of synchronous CBC, the lesion in the breast that was first suspected of cancer during clinical or imaging examinations was defined as the first breast cancer. Subsequent lesions that were found in the contralateral breast on MRI were referred to as the contralateral cancer [23–25]. Study data such as post-menopausal status were determined by the database of clinical medical records.

### MRI protocol

All breast MRI examinations were performed using a 3.0 T scanner (MAGNETOM Verio, Siemens Healthcare, Erlangen, Germany). Patients were placed in the prone position, with imaging acquired using a dedicated breast coil. The scanning protocol was consistent with international recommendations [26].The imaging protocol consisted of a localization sequence and an axial T2-weighted short-tau inversion-recovery sequence (TR = 3770 ms, TE = 69 ms, slice thickness = 4 mm), a T1-weighted non–fat-suppressed MR sequence (TR = 260 ms, TE = 25 ms, slice thickness = 3 mm), and a dynamic contrast-enhanced sequence (TR = 4.7 ms, TE = 1.7 ms, slice thickness = 1.5 mm). For dynamic contrast-enhanced T1-weighted axial fat-suppressed imaging, a single pre-contrast sequence and six post-contrast sequences were acquired following intravenous injection of gadopentetate dimeglumine(Jiangsu Hengrui Pharmaceutical Co., Ltd., China). The contrast agent was administered at a dose of 0.1 mmol per kilogram of body weight, and the temporal resolution of each dynamic acquisition was 59 s.After MRI examination, subtraction MRI images were generated by subtracting pre-contrast T1-weighted images from the first to sixth post-contrast dynamic phases of the T1-weighted sequence.

For premenopausal women, MRI examinations were performed on Days 7–14 of their menstrual cycle (Day 1 defined as the first day of menstruation). For postmenopausal women, MRI examinations were scheduled according to patient-confirmed appointment times [7].

### Image analysis

Two breast imaging radiologists (26 and 11 years of breast imaging experience, respectively) reviewed the MRI scans independently. They were blinded to the pathological results of the study group and the control group. They recorded the BPE level and FGT amount. Evaluations were performed independently by the two readers, and no consensus process was employed, to better mirror routine clinical practice and capture the full spectrum of interpretive variability.

**Fig. 1:**
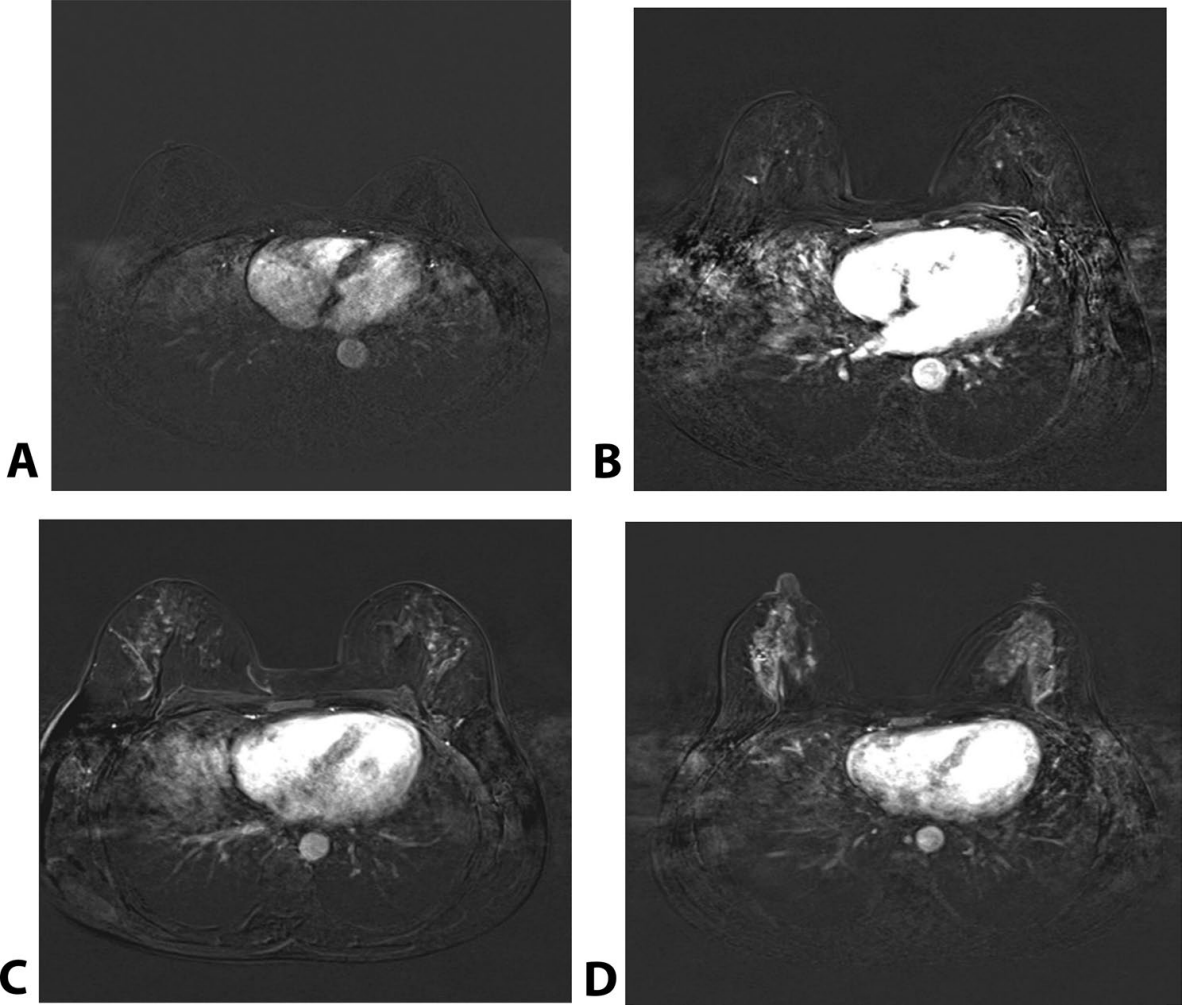
T1-weighted fat-suppressed first contrast-enhanced subtraction MRI scans depicting different levels of BPE (**A**) Minimal BPE, (**B**) mild BPE, (**C**) moderate BPE, and (**D**) marked BPE

**Fig. 2:**
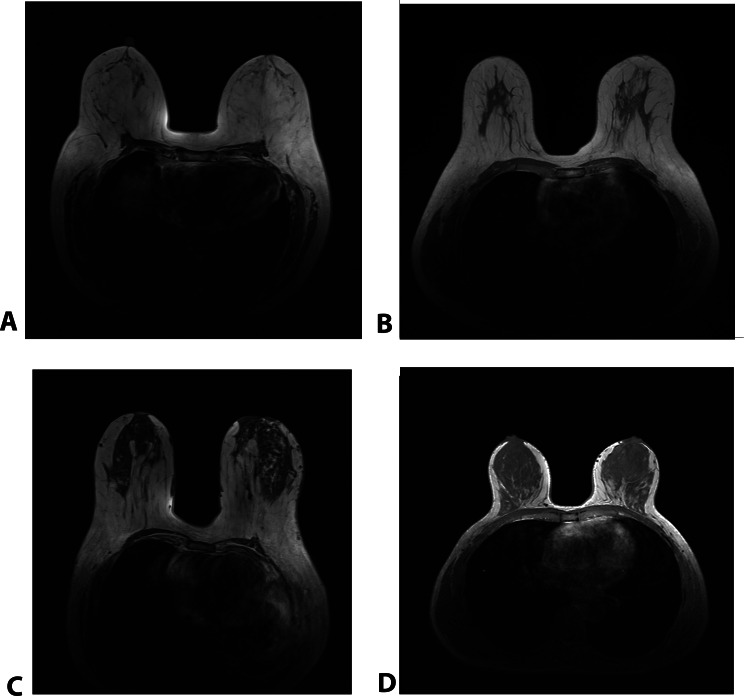
T1-weighted non–fat-suppressed MRI scans depicting different levels of FGT. (**A**) Almost entirely fatty, (**B**) scattered areas of fibroglandular tissue, (**C**) heterogeneous fibroglandular tissue, and (**D**) extreme fibroglandular tissue

The level of BPE was visually evaluated from the fat-suppressed T1-weighted pre-contrast and first post-contrast and subtraction images [18]. In the study group, in order to obtain more reliable results, images of the contralateral lesion-free breast were selected for BPE evaluation; for multifocal lesions, lesion-free slices from bilateral breast images were selected for comprehensive evaluation. The BPE level was categorized as minimal, mild, moderate, and marked according to the BI-RADS criteria of the American Academy of Radiology in 2013 [18] (Fig. 1). The amount of FGT was visually evaluated from the T1-weighted non–fat-suppressed sequence and was categorized as almost entirely fatty, scattered areas of FGT, heterogeneous FGT, or extreme FGT according to the criteria of the American Academy of Radiology in 2013 [18] (Fig. 2).

### Statistical analysis

The unit of statistical analysis in this study was the patient rather than the lesion to avoid pseudoreplication in multifocal cases. The balance between the two groups was assessed using absolute standardized mean differences (|SMD|). Univariate conditional logistic regression was used to analyze the ORs and corresponding 95% confidence intervals of BPE and FGT in relation to synchronous CBC, with each variable analyzed separately [22]. Owing to the small sample sizes in individual ordinal categories, we combined the lower and higher categories of the four-point variables BPE and FGT to generate more accurate and stable estimations.The minimal and mild BPE combined as the lower categories for BPE, the moderate and marked BPE combined as the higher categories for BPE. The almost entirely fat and Scattered fibroglandular combined as the lower categories for FGT, and the heterogeneous and extreme fibroglandular combined as the higher categories for FGT. In order to reduce the impact of menstrual cycle and endogenous hormones on BPE, we conducted a separate analysis on postmenopausal women. By using ROC analysis, the factors with significant differences between the study group and the control were further analyzed to determine the AUC, sensitivity and specificity. The scores assigned by readers 1 and 2 were statistically analyzed independently. The DeLong test was used to compare whether there was a statistically significant difference in the AUC between Reader 1 and Reader 2.Kappa analysis was used to calculate inter-reader interpretation. The agreement based on the kappa value was defined as follows: 0.00–0.20, slight agreement; 0.21–0.40, fair agreement; 0.41–0.60, moderate agreement; 0.61–0.81, substantial agreement; 0.81–1.00, almost perfect agreement. Post-hoc power analyses for BPE and FGT were performed based on the conditional logistic regression model, with effect sizes (Cohen’s f²) set according to the observed ORs in this study, and a power (1-β) ≥ 80% considered sufficient.

*P* < 0.05 was considered to indicate statistical significance [27].All statistical tests were conducted using SPSS 25.0 (International Business Machines Corporation, Armonk, New York, USA).

## Results

### Characteristics of the study and control group

The characteristics of the study group (*n* = 61) and the control group (*n* = 122) are shown in Table 1. Baseline characteristics were balanced between the two groups. All covariates in this study exhibited |SMD| values < 0.20, confirming that the baseline balance met the preset criteria (Table 1).

**Table 1:** Characteristics of the synchronous CBC study group and UBC control group

Characteristics	Synchronous CBC (*n* = 61)	UBC (*n* = 122)	Z	*P*	SMD
Median age (y)	63	62	0.190	0.850^a^	0.021
Age range (y)	42–85	43–85			
Menopausal status			-	0.818^b^	
Premenopausal	7 (11%)	16 (13%)			-0.081
Postmenopausal	54 (89%)	106 (87%)			
Histologic subtype ^c^			-	1.000^b^	
Ductal	56 (92%)	113 (93%)			-0.056
Other ^d^	5 (8%)	9 (7%)			
Carcinoma in situ or invasive carcinoma			-	0.527^b^	
carcinoma in situ	8 (13%)	21 (17%)			-0.175
invasive carcinoma	53 (87%)	101 (83%)			
ER status ^e^			-	0.066^b^	
ER+	43 (70%)	93 (76%)			-0.175
ER-	12 (20%)	27 (22%)			
Unknown	6 (10%)	2 (2%)			

Abbreviations: CBC: contralateral breast cancer; UBC: unilateral breast cancer

^a^ Mann–Whitney *U*-test

^b^ Fisher exact test(the exact probability method)

c Histologic subtype refers to the first breast cancer in patients with synchronous breast cancer

^d^ Other refers to invasive lobular, invasive mucinous carcinoma, and invasive tubular carcinoma

^e^ ER status refers to estrogen receptor status of the first breast cancer in patients with synchronous breast cancer

Note: The balance of both quantitative and qualitative data between the two groups was evaluated using the standardized mean difference (SMD).|SMD| < 0.10: Excellent balance;0.10 ≤ |SMD| < 0.20: Acceptable balance;

|SMD| ≥ 0.20: Imbalanced

In both the study group (*n* = 61) and control group (*n* = 122), the surgery was performed within 3 weeks after MRI examination. Among the total 61 patients with bilateral lesions of the contralateral carcinoma in the study group, 48 had bilateral breast lesions removed together in one anesthesia, while the other 13 had bilateral lesions removed in two surgeries with intervals no more than two weeks according to the database. The age range of patients in the study group was 42–85 years old, with a median age of 63 years. Among the 61 patients with synchronous CBC, 53 (87%) had invasive carcinoma, 8 (13%) had carcinoma in situ without an invasive component. The histological subtypes identified were ductal carcinoma in 56 (92%) cases and other subtypes in 5 (8%) cases. Among the contralateral breast lesions in 61 patients with synchronous CBC, 16 (26%) were ductal carcinomas in situ, 31 (51%) were invasive ductal carcinomas, 5 (8%) were invasive lobular carcinomas, 1 (2%) was lobular carcinoma in situ, 4 (7%) were papillary carcinoma, 2 (3%) were invasive tubular carcinoma, 1 (2%) was mucinous carcinoma, and 1 (2%) was squamous cell carcinoma. The age range of patients in the control group was 43–85 years old, with a median age of 62 years.Among the 122 patients with UBC, 101 (83%) had invasive carcinoma, 21 (17%) had carcinoma in situ without an invasive component. The histological subtypes identified were ductal carcinoma in 113 (93%) cases and other subtypes in 9 (7%) cases. Other pathological sub-types included lobular carcinoma, mucinous carcinoma and tubular carcinoma.

A total of 70 lesions were identified in the contralateral breasts of the study group, including 4 patients with multifocal lesions. Among 70 lesions, 53 lesions presented as masses, and 17 lesions presented as non-mass enhancement on MRI.The MRI features of the mass lesions were diverse, with a median size of 11.90 mm (range: 3–25.80 mm). All the non-mass enhancement lesions were identified from BPE on first phase post-contrast axial images. Their histological types were ductal carcinoma, 9 cases were ductal carcinoma in situ and 8 cases were invasive carcinoma. In all 17 cases, their distribution patterns were focal in 7 cases, linear in 5 cases, segmental in 4 cases and regional in 1 case. The internal enhancement patterns were heterogeneous in 14 cases and homogeneous in 3 cases. There were 8 cases with typical clumped or cluster ring patterns.

### Association of qualitative MRI features with synchronous CBC

Conditional logistic regression showed that the BPE level was associated with synchronous CBC in patients with newly diagnosed breast cancer (Reader 1: *P* < 0.01, Reader 2: *P* < 0.01). Compared with patients with minimal BPE, patients with mild BPE showed a significantly higher likelihood of synchronous CBC (Reader 1: OR = 2.67, Reader 2: OR = 3.74), as did those with moderate BPE (Reader 1: OR = 5.23, Reader 2 = 7.75) and marked BPE (reader 1: OR = 3.14, reader 2: OR = 5.12).

Consistent trends were observed when BPE was analyzed as a dichotomous variable. Compared with women with minimal or mild BPE, women with moderate or marked BPE were more likely to have synchronous CBC (Reader 1: OR = 2.90, *P* < 0.01; Reader 2: OR = 3.36, *P* = 0.02). Given the strong relationship of BPE with the menstrual cycle and endogenous hormones, we analyzed postmenopausal women separately, and the results did not change (Table 2). Supplementary verification of conditional logistic regression assumptions (for both BPE and FGT analyses) confirmed no abnormal influential points (leverage values & Cook’s distance), no multicollinearity (all VIF < 5), and good model fit (Hosmer-Lemeshow test, *P* > 0.05).

**Table 2:** Association of qualitative BPE level with synchronous CBC

BPE level	Synchronous CBC (*n* = 61)	UBC (*n* = 122)	OR	95% CI	*P* value
Reader 1					
Minimal(control)	20	77	-	-	< 0.01
Mild	27	32	2.67	1.38–5.14	
Moderate	10	7	5.23	1.69–16.21	
Marked	4	6	3.14	0.77–12.78	
Reader 2					
Minimal(control)	12	65	-	-	< 0.01
Mild	33	41	3.74	1.75–7.96	
Moderate	11	9	7.75	2.21–27.13	
Marked	5	7	5.12	1.18–22.15	
**dichotomous**					
Reader 1					
Minimal or Mild(control)	47	109	-	-	< 0.01
Moderate or Marked	14	13	2.90	1.14–7.41	
Reader 2					
Minimal or Mild(control)	45	106	-	-	0.02
Moderate or Marked	16	16	3.36	1.25–9.01	
**Postmenopausal subgroup**					
Reader 1					
Minimal or Mild(control)	44	99	-	-	0.02
Moderate or Marked	10	7	4.13	1.27–13.42	
Reader 2					
Minimal or Mild(control)	42	97	-	-	0.01
Moderate or Marked	12	9	5.75	1.56–21.20	

Abbreviations: CBC: contralateral breast cancer; UBC: unilateral breast cancer, BPE: background parenchymal enhancement; CI: confidence interval

Post-hoc power analysis showed the statistical power for this association, with values of 0.967 (Reader 1) and 0.986 (Reader 2). In contrast, no significant association was observed between the amount of FGT and synchronous CBC (Reader 1: *P* = 0.94; Reader 2: *P* = 0.71) (Table 3).The null association remained consistent when FGT was analyzed as a dichotomous variable, indicating that FGT may not correlate with synchronous CBC risk in this cohort. Corresponding post-hoc power analysis showed the statistical power values of 0.963 (Reader 1) and 0.982 (Reader 2).

**Table 3:** Association of qualitative FGT amount with synchronous CBC

Amount of FGT	Synchronous CBC (*n* = 61)	UBC (*n* = 122)	OR	95% CI	*P* value
Reader 1	…	…			
Almost entirely fat(control)	12	26	-	-	0.94
Scattered fibroglandular	21	43	1.05	0.45–2.47	
Heterogeneous fibroglandular	24	43	1.20	0.51–2.86	
Extreme fibroglandular	4	10	0.84	0.19–3.76	
Reader 2					
Almost entirely fat(control)	7	20	-	-	0.71
Scattered fibroglandular	18	38	1.38	0.50–3.82	
Heterogeneous fibroglandular	30	50	1.70	0.64–4.49	
Extreme fibroglandular	6	14	1.23	0.33–4.56	
**FGT (dichotomous)**					
Reader 1					
Almost entirely fat or scattered(control)	33	69	-	-	0.75
Heterogeneous or extreme fibroglandular	28	53	1.11	0.59–2.09	
Reader 2					
Almost entirely fat or scattered(control)	25	58	-	-	0.41
Heterogeneous or extreme fibroglandular	36	64	1.29	0.70–2.37	
**Postmenopausal subgroup**					
Reader 1					
Almost entirely fat or scattered(control)	32	65	-	-	0.74
Heterogeneous or extreme fibroglandular	22	41	1.11	0.59–2.12	
Reader 2					
Almost entirely fat or scattered(control)	24	56	-	-	0.30
Heterogeneous or extreme fibroglandular	30	50	1.39	0.75–2.56	

Abbreviations: CBC: contralateral breast cancer; UBC: unilateral breast cancer; FGT: fibroglangular tissue; CI: confidence interval

**Fig. 3:**
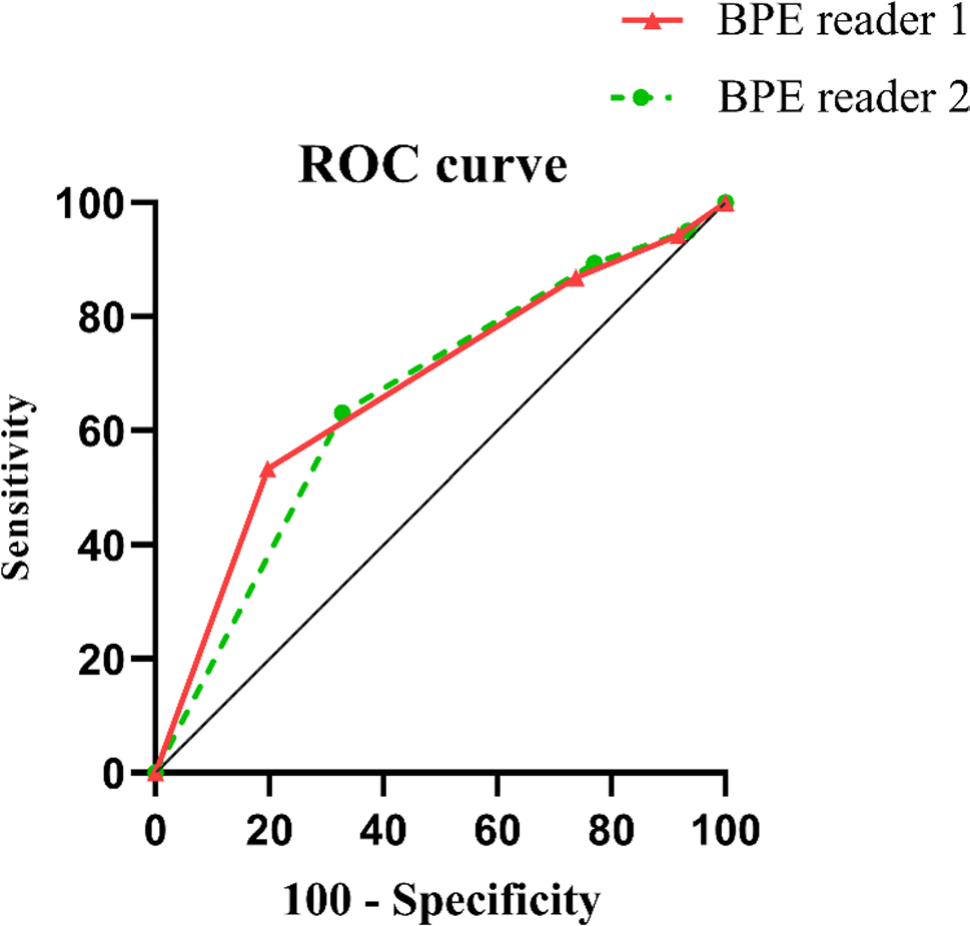
ROC showed the accuracy of BPE level in differentiating synchronous CBC patients from control subjects

The ROC determined that the optimal threshold was greater than minimal BPE. Patients with mild, moderate or marked BPE showed a higher likelihood of having synchronous CBC than patients with minimal BPE. The AUC was 0.66 (95% CI: 0.57–0.74) with sensitivity and specificity of 67.2% (95% CI: 0.54–0.78) and 63.1% (95% CI: 0.54–0.72) respectively by reader1. The AUC was 0.67 (95% CI: 0.59–0.76) with sensitivity and specificity of 80.3% (95% CI: 0.68–0.89) and 53.3% (95% CI: 0.44–0.62) respectively by reader2 (Fig. 3). The DeLong test indicated no statistically significant difference in the AUC between Reader 1 and Reader 2 (95% CI: -0.03–0.06; Z = 0.823, *P* = 0.410). There was substantial agreement between Reader 1 and Reader 2 with regard to qualitative assessment of BPE level (K = 0.75, *P* < 0.01) and amount of FGT (K = 0.72, *P* < 0.01).

## Discussion

The present study explored whether BPE levels and FGT amount were associated with synchronous CBC in patients with UBC undergoing preoperative MRI. The preliminary findings demonstrated a correlation between high BPE level on MRI and the presence of synchronous CBC in newly diagnosed breast cancer patients. However, the amount of FGT on MRI was not associated with synchronous CBC. To the best of our knowledge, the association between BPE level, the amount of FGT and synchronous CBC risk has received little attention in the literature. This study provides preliminary evidence of an association between BPE and synchronous CBC in patients with newly diagnosed breast cancer. However, without adjustment for key potential confounders, it remains unclear whether this association reflects a true effect of BPE or is driven by residual confounding. The findings should therefore be interpreted as strictly hypothesis-generating.

Previous studies have confirmed that BPE is a predictor of breast cancer in high-risk populations [11, 12]. Notably, emerging evidence suggests that for women with a personal history of breast cancer, BPE on breast MRI is an independent predictive factor for the subsequent development of a second breast cancer (including CBC) [28].Building on these findings, our results further demonstrate that high BPE levels are useful for identifying a potential association with synchronous CBC in patients with unilateral cancer. A recent publication reported that contralateral breast prophylactic irradiation can reduce the occurrence or delay CBC in BRCA-positive patients who undergo early-stage breast cancer treatment [29]. Another study showed that its mechanism may be due to the reduction of contralateral breast BPE level [14]. While the two aforementioned studies differ from ours in terms of patient characteristics, their findings also indicate that lower BPE levels correlate with a decreased risk of contralateral breast cancer.

No association was found between synchronous CBC and the amount of FGT in the present study. Consistent with this finding, Dontchos et al. reported no correlation between FGT and the risk of UBC in their research on UBC risk factors, while identifying an association with BPE(OR = 1) [30]. Additionally, it has been shown that the BPE level changes significantly after bilateral salpingo-oophorectomy, while FGT changes take more time to show [20].Furthermore, other evidence indicates that BPE is a more sensitive indicator of breast cancer risk than FGT [31]. Some other studies have also indicated that mammographic density and FGT are not related to BPE [9]. These findings together may indicate differences in the carcinogenic mechanisms of BPE and FGT, but this needs to be explored in future studies.

At present, there is no study on the pathological mechanism of BPE in synchronous CBC. The existing articles on the pathophysiological mechanism of BPE show that: as a biomarker of breast tissue, BPE indirectly reflects the microenvironment of breast tissue, and changes in the microenvironment is key to promoting malignant transformation [32]. A study indicated that BPE is highly correlated with microvessel density and CD34 levels in breast glandular tissue in premenopausal patients [33]. Further, preliminary data show that in women with higher BPE, the breast is characterized by greater glandular tissue concentration, greater metabolic activity, greater vascularity, and changes in hormone levels [1, 34].Some previous studies have suggested that the microenvironment of the tumor within a host is a key factor in the pathogenesis of synchronous CBC, and its carcinogenic mechanism may be different from that of UBC [35]. However, the exact biological mechanism underlying elevation in BPE and the relationship between the level of BPE and the microenvironment of the breast tumor in patients with synchronous bilateral breast cancer need further study.

Timely identification of the risk of synchronous CBC holds significant clinical value. It facilitates the optimization of screening workflows, enabling the implementation of bilateral breast MRI for high-risk populations to detect occult contralateral lesions, while also supporting the development of individualized treatment plans. Furthermore, early risk stratification refines prognostic assessment and reduces the risk of disease recurrence. However, the risk predictive ability of BPE reported in this study—with an area under the curve (AUC) of 0.66–0.67—is relatively modest. This indicates that while BPE holds some predictive value as an independent risk factor, it is insufficient to guide clinical diagnosis and treatment when used in isolation.

This study has several limitations. First and foremost, we could not adjust for key BPE/CBC-related confounders, such as genetic risk and mammographic density. This residual confounding may mean the observed association between BPE and synchronous CBC is due to confounding rather than a causal relationship, so our results should be considered hypothesis-generating only.However, our sensitivity analyses may help to support the robustness of this association, potentially reducing the chance of bias caused by unmeasured confounders or model specification issues. Second, the premenopausal subgroup had an extremely small sample size (7 cases/16 controls), which severely limited the reliability of its findings. Indeed, our stratified (sensitivity) analysis confirmed that this subgroup had an exceedingly low post-hoc power, indicating that the results are unstable and likely exploratory. In contrast, the association remained significant in the adequately powered postmenopausal subgroup, suggesting the main finding is not dependent on the underpowered premenopausal analysis.Third, the assessment of BPE and FGT in this study relied entirely on qualitative analysis based on the BI-RADS classification criteria. It should be explicitly acknowledged that MRI image interpretation is inherently subjective, which may lead to interobserver variability and thereby limit the reproducibility of the results.In this study, we adopted independent reader assessments without consensus, a practice that helps avoid group bias and better simulates real-world clinical settings where single-reader interpretation is common, though it may introduce random measurement error. However, the substantial inter-reader agreement (K = 0.75) observed suggests that such error is unlikely to have substantially weakened the reported associations.Currently, there is no unified standardized quantitative method for BPE evaluation, and existing quantitative approaches vary significantly [36, 37]. Future research should focus on standardizing quantitative BPE assessment methods and validating their clinical application, thereby reducing subjective bias and improving the reliability of research findings [38].Finally, it should be emphasized that multiple statistical tests were conducted in this study without correcting for multiple comparisons, which may potentially inflate the risk of Type I error (i.e., false-positive results), and this should be taken into account when interpreting the study conclusions.

## Conclusion

Based on the present study’s findings, a high BPE level correlates with an elevated risk of synchronous CBC, indicating a potential association that does not imply causality or independence.This correlation may be subject to residual confounding, and thus BPE alone cannot support clinical decision-making.

## Supplementary Information

Below is the link to the electronic supplementary material.

Supplementary Material 1

## Data Availability

The data underlying this article will be shared on reasonable request to the corresponding author.
