# Supplementary material for: The relationship between background parenchymal enhancement, amount of fibroglandular tissue and synchronous contralateral breast cancer in preoperative magnetic resonance imaging of newly diagnosed breast cancer patients
Source: BMC Med Imaging. 2026 May 20;26:346. doi: 10.1186/s12880-026-02402-3 (PMC13366652; doi:10.1186/s12880-026-02402-3)
Supplement: Supplementary file 1 — Supplementary Material 1 [file 12880_2026_2402_MOESM1_ESM.docx]

Supplementary Material Sensitivity Analysis (1-4): Unmatched Multivariable Logistic Regression, Stratified Analysis, Ordinal Variable Analysis and Stability Checks

To validate the robustness of the associations between breast parenchymal enhancement (BPE), fibroglandular tissue (FGT), and contralateral breast cancer (CBC), the following sensitivity analyses were conducted in accordance with the reviewers’ recommendations. All statistical analyses were performed using SPSS 25.0 (International Business Machines Corporation, Armonk, New York, USA). Detailed methodologies and corresponding results are described below.

1. Unmatched Multivariable Logistic Regression

Methods:An unmatched multivariable logistic regression model was constructed, with contralateral breast cancer (CBC, present vs. absent) as the dependent variable. Candidate covariates included menopausal status, estrogen receptor (ER) status, BPE, FGT, histological type (ductal carcinoma vs. other subtypes), and tumor infiltration status (in situ carcinoma vs. invasive carcinoma). Separate analyses were performed based on BPE assessments by two independent readers (reader 1 and reader 2), using the forward likelihood ratio (LR) method for variable selection (entry criterion: P<0.05; removal criterion: P>0.10). Missing data were handled by listwise deletion, and multicollinearity was excluded by verifying that the variance inflation factor (VIF) was <2.0 for all variables.

Results: In the final model, BPE was the only variable retained, showing a potential association with CBC. Other covariates (menopausal status, ER status, FGT, histological type, infiltration depth) were not retained during stepwise selection (all P> 0.10), suggesting they may not have independent effects on CBC in this model. Details are in Supplementary Table 1.

Supplementary Table 1.Association of qualitative BPE level with synchronous CBC

| BPE level | Synchronous CBC  (n = 61) | UBC  (n= 122) | OR | 95% CI | P value |
| --- | --- | --- | --- | --- | --- |
| Reader 1 | | | | | |
| Minimal(control) | 20 | 77 | - | - | 0.001 |
| Mild | 27 | 32 | 3.75 | 1.79-7.86 |  |
| Moderate | 10 | 7 | 5.11 | 1.63-16.02 |  |
| Marked | 4 | 6 | 2.98 | 0.76-11.73 |  |
| Reader 2 | | | | | |
| Minimal(control) | 12 | 65 | - | - | <0.001 |
| Mild | 33 | 41 | 4.80 | 2.12-10.78 |  |
| Moderate | 11 | 9 | 7.11 | 2.32-21.80 |  |
| Marked | 5 | 7 | 4.57 | 1.21-17.24 |  |

Abbreviations: CBC: contralateral breast cancer; UBC: unilateral breast cancer, BPE: background parenchymal enhancement; FGT: fibroglangular tissue; CI: confidence interval

2: Stratified Analysis by Menopausal Status​

Methods:Stratified analysis was performed according to menopausal status (premenopausal vs. postmenopausal) to investigate whether the associations of BPE and FGT with synchronous contralateral breast cancer (CBC) differed by menopausal status. The postmenopausal subgroup had already been analyzed in the main manuscript. Given the extremely small sample size of the premenopausal subgroup, an independent analysis was conducted specifically for this subgroup. Additionally, post-hoc statistical power was recalculated for the premenopausal subgroup to assess the reliability and interpretability of the subgroup-specific results.

Results: Independent analysis of the premenopausal subgroup demonstrated no significant associations between either BPE or FGT and synchronous CBC. Given the extremely limited sample size of this subgroup, recalculated post-hoc statistical power revealed that the statistical power for both BPE- and FGT-related analyses was below 20%. This extremely low statistical power mandates extreme caution in the interpretation of the premenopausal subgroup results; these findings are strictly exploratory in nature and do not possess any confirmatory reference value. Detailed stratified results and power analysis data are provided in Supplementary Table S2-3.

Supplementary Table 2.

Association of qualitative BPE level with synchronous CBC for premenopausal subgroup

| Premenopausal subgroup | Synchronous CBC  (n = 61) | UBC  (n= 122) | OR | 95% CI | P value |
| --- | --- | --- | --- | --- | --- |
| **BPE level** | | | | | |
| Reader 1 |  |  |  |  |  |
| Minimal or Mild(control) | 3 | 10 | - | - | 0.58 |
| Moderate or Marked | 4 | 6 | 1.65 | 0.29–9.51 |  |
| Reader 2 |  |  |  |  |  |
| Minimal or Mild(control) | 3 | 9 | - | - | 0.76 |
| Moderate or Marked | 4 | 7 | 1.34 | 0.21–8.47 |  |
| **Amount of FGT** | | | | | |
| Reader 1 |  |  |  |  |  |
| Almost entirely fat or scattered(control) | 1 | 4 | - | - | 0.81 |
| Heterogeneous or extreme fibroglandular | 6 | 12 | 0.71 | 0.04–11.79 |  |
| Reader 2 |  |  |  |  |  |
| Almost entirely fat or scattered(control) | 1 | 2 | - | - | 0.81 |
| Heterogeneous or extreme fibroglandular | 6 | 14 | 0.71 | 0.04–11.79 |  |

Abbreviations: CBC: contralateral breast cancer; UBC: unilateral breast cancer, BPE: background parenchymal enhancement; FGT: fibroglangular tissue; CI: confidence interval

Supplementary Table 3. Post-hoc Power Analysis

| Analysis Target / Reader | Statistical Power (1-β) |
| --- | --- |
| Overall -BPE（reader1） | 0.967 |
| Overall -BPE（reader2） | 0.986 |
| Premenopausal subgroup（reader1） | 0.162 |
| Premenopausal subgroup（reader2） | 0.141 |
| Overall -FGT（reader1） | 0.963 |
| Overall -FGT（reader2） | 0.982 |
| Premenopausal subgroup（reader1） | 0.096 |
| Premenopausal subgroup（reader2） | 0.181 |

3.Ordinal Variable Analysis

Methods: To address potential small-sample bias and obtain more robust estimates, Firth penalized logistic regression was performed, with BPE treated as a 4-level ordinal variable (minimal, mild, moderate, marked). The primary objective was to evaluate the association between incremental increases in BPE levels and the risk of synchronous contralateral breast cancer (CBC). Separate analyses were conducted based on BPE assessments by two independent readers (reader 1 and reader 2).

Results: Firth penalized regression results demonstrated that each incremental increase in BPE level was significantly associated with an elevated risk of synchronous CBC. Specifically, for each one-level advancement in the BPE ordinal scale (from minimal to mild, mild to moderate, or moderate to marked), the risk of synchronous CBC increased by 2.08-fold (reader 1) to 2.36-fold (reader 2). The application of Firth's penalized likelihood approach effectively mitigated potential small-sample bias, thereby providing more reliable and robust estimates for the study dataset. Detailed results are provided in Supplementary Table 4.

Supplementary Table 4 Association of qualitative BPE level with synchronous CBC

|  | OR | P value | OR 95% CI |
| --- | --- | --- | --- |
| BPE (Reader 1) Firth Penalized Regression / BPE | 2.08 | 0.014 | 1.40-3.19 |
| BPE (Reader 2) Firth Penalized Regression / BPE | 2.36 | <0.001 | 1.57-3.70 |

## Stability Checks

## Methods: A stability check was performed using the “leave-one-matching-variable-out” approach to assess result stability. Repeated unmatched multivariable logistic regression (forward LR: entry P<0.05, removal P>0.10) was conducted, excluding one matching variable each time. Matching variables: age (continuous), histological type (ductal vs. others), and infiltration depth (in situ vs. invasive).

Results: The three models (each excluding one matching variable) yielded identical results. In all models, BPE was consistently strongly associated with CBC, with highly consistent adjusted OR values, P-values, and 95% CIs. Excluding any matching variable did not change the core finding that higher BPE correlates with increased CBC risk, indicating good stability of the association.
